# Comparison of different vascular risk engines in the identification of type 2 diabetes patients with high cardiovascular risk

**DOI:** 10.1186/s12872-015-0120-3

**Published:** 2015-10-13

**Authors:** Antonio Rodriguez-Poncelas, Gabriel Coll-de-Tuero, Marc Saez, José M. Garrido-Martín, José M. Millaruelo-Trillo, Joan Barrot de-la-Puente, Josep Franch-Nadal, on behalf RedGDPS Study Group

**Affiliations:** Primary Healtcare Center (PHC) Anglès, Girona, Spain; Unitat de Suport a la Recerca Barcelona Ciutat, Institut Universitari d’Investigació en Atenció Primària Jordi Gol (IDIAP Jordi Gol), Barcelona, Spain; Department of Medical Sciences, University of Girona, Girona, Spain; Research Group in Statistic,Applied economy and Health. (GRECS), University of Girona, Girona, Spain; PHC Torrero La Paz, Zaragoza, Spain; PHC Salt, ICS, Salt, Girona, Spain; PHC Raval Sud, ICS, Barcelona, Spain; Research Unit, IdIAP, Maluquer Salvador,11, 17002 Girona, Spain

**Keywords:** Type 2 diabetes, Cardiovascular risk prediction, Cardiovascular disease, Risk prediction models, Primary prediction

## Abstract

**Background:**

Some authors consider that secondary prevention should be conducted for all DM2 patients, while others suggest that the drug preventive treatment should start or be increased depending on each patient’s individual CVR, estimated using cardiovascular or coronary risk functions to identify the patients with a higher CVR. The principal objective of this study was to assess three different cardiovascular risk prediction models in type 2 diabetes patients.

**Methods:**

Multicentre, cross-sectional descriptive study of 3,041 patients with type 2 diabetes and no history of cardiovascular disease. The demographic, clinical, analytical, and cardiovascular risk factor variables associated with type 2 diabetes were analysed. The risk function and probability that a cardiovascular disease could occur were estimated using three risk engines: REGICOR, UKPDS and ADVANCE. A patient was considered to have a high cardiovascular risk when REGICOR ≥ 10 % or UKPDS ≥ 15 % in 10 years or when ADVANCE ≥ 8 % in 4 years.

**Results:**

The ADVANCE and UKPDS risk engines identified a higher number of diabetic patients with a high cardiovascular risk (24.2 % and 22.7 %, respectively) compared to the REGICOR risk engine (10.2 %). The correlation using the REGICOR risk engine was low compared to UKPDS and ADVANCE (*r* = 0.288 and *r* = 0.153, respectively; *p* < 0.0001). The agreement values in the allocation of a particular patient to the high risk group was low between the REGICOR engine and the UKPDS and ADVANCE engines (*k* = 0.205 and *k* = 0.123, respectively; *p* < 0.0001) and acceptable between the ADVANCE and UKPDS risk engines (*k* = 0.608).

**Conclusions:**

There are discrepancies between the general population and the type 2 diabetic patient-specific risk engines. The results of this study indicate the need for a prospective study which validates specific equations for diabetic patients in the Spanish population, as well as research on new models for cardiovascular risk prediction in these patients.

## Background

Type 2 diabetes mellitus (DM2) is associated with an increased risk of cardiovascular disease (CVD) [1–3]. This risk is two to four times higher in DM2 patients than in non-DM2 patients, independently of the classical cardiovascular risk factors (CVRF). Incidence of diabetes is expected to continue to increase over in the following years and CVD associated with DM2 will most likely increase as well [4]. Although diabetes is a risk factor for Coronary Heart Disease (CHD), whether diabetes alone is a CHD equivalent in assessing de risk of future CVD events is controversial. It might be related to multiple causes, such as those attributable to the different baseline characteristics and different risk profiles of the diabetic patients who participated in the studies [5, 6]. Type 2 diabetes patients without other CVRF have a lower risk of CVD, especially patients with a shorter evolution of the disease and with fewer metabolic alterations [7]. According to some studies, the age of diagnosis and the time course of the disease play a role in its progression, and after 10 to 15 years, the coronary risk of these diabetic patients is similar to patients with prior CHD [6, 8, 9].

Some authors consider that secondary prevention should be conducted for all DM2 patients [10–12], while others suggest that the drug preventive treatment should start or be increased depending on each patient’s individual CVR, especially in countries with limited health resources, and recommend using cardiovascular or coronary risk functions to identify the patients with a higher CVR who could benefit more from a treatment scale-up. Moreover, overtreatment in patients at low risk might be associated with a higher risk of drug interactions, more significant cost, inconvenience, and side effect burdens that might engender higher rates of non-adherence [13, 14]. Clearly, these risk functions should not be applied to DM2 patients with a previous CVD.

In Spain, several risk strategies are currently used to estimate CVR. Among these equations, Framingham-REGICOR (Registre Gironí del Cor) [15] and SCORE (Systematic Coronary Risk Evaluation) [16] are the most popular. It has been discussed that the former overestimates and the second underestimates CVR [17]. Other risk models, such as the UK Prospective Diabetes Study (UKPDS) [18] and the Action in Diabetes and Vascular Disease Preterax and Diamicron MR Controlled Evaluation (ADVANCE) [19], have been developed exclusively on a DM2 patient cohort.

The ultimate aim of this study was to compare three different cardiovascular risk-prediction models in three diabetic patient-based cohorts to determine the variables involved in the identification of high risk patients, according to the equation used, and to analyse the variables associated with the discrepancy among the three risk functions.

## Methods

Cross-sectional descriptive study. Patients were selected from the database of three previous studies of the Network of Diabetes Study Group in Primary Health Care (redGDPS, for its initials in Spanish), which were: CVRF-Primary Health Care Diabetes Study Group (GEDAPS); Study of Immigrant Diabetes in Spain (IDIME) and Prevalence of chronic kidney disease in patients with type 2 diabetes in Spain (PERCEDIME2). The methodology, design and results of these studies have been published previously [20–22]. In the three studies the following methodology had been used. Health providers were instructed to obtain a random sample from the medical records of type 2 DM patients with a follow-up greater than 6 months since diagnosis. A total sample of five patients multiplied by the number of basic care units, with a minimum of 30 patients per centre, was required. A pre-selection of medical records with an additional 20 % was performed. Data were collected from paper medical records from 1997 to 2002 and from electronic records in 2007 (FRCV-GEDAPS). A multicentre, observational, cross-sectional study including a cohort of 605 diabetic immigrants and 307 native diabetics was conducted on patients diagnosed with diabetes and treated in primary and specialised care in Spain. A consecutive sampling method had been followed (IDIME). Finally, a national cross-sectional study was performed in primary care consults with 1,145 type 2 diabetics. The patients included both genders and they were over 40 years old. Moreover, the patients had all of the variables necessary for the study included in their clinical history. The patients were selected by a convenience sample of the first three patients with DM2 who came to the consultation each day for any reason and met the inclusion criteria until the number of participants per researcher was completed. The information for each participant was collected in the period from February to July 2011 (PERCEDIME2). FRCV-GEDAPS study was approved by the Consell Assessor de la Diabetes (Advisory Board on Diabetes) of the Health Departament of Catalunya; IDIME study was approved by the Clinical Research Ethics Committee of the IMIM-Hospital del Mar, Barcelona, Spain (Project number 2008/3176/I) and PERCEDIME2 study was approved by the Ethical and Clinical Investigation Committee of the Institut d’Assistència Sanitària (IAS), Girona, Spain. All participants provided written informed consent.

### Analysed variables

The following variables were analysed: age, gender, ethnicity, years since diabetes diagnosis, %HbA1c, body mass index (BMI) kg/m [2], waist circumference (WC) in cm; tobacco consumption (considering a daily consumer of any amount as a smoker and as an ex-smoker after one year of abstinence); systolic (SBP) and diastolic blood pressure (DBP) and pulse pressure (PP) (mmHg), including the diagnosis of high blood pressure (3 readings ≥ 140/90 mmHg); total cholesterol (mg/dl), HDL cholesterol (mg/dl), and non-HDL cholesterol (mg/dl), as well as hypercholesterolemia criteria (2 values ≥ 250 mg/dl); diabetes mellitus according to the American Diabetes Association [23], as well as previously diagnosed cases or being on anti-hyperglycaemic treatment prior to the study; glomerular filtrate (GF) measure using the MDRD-4 equation [24] and urine albumin excretion rate according to albumin/creatinine ratio (mg/g).

The possibility of presenting a CVD was estimated using three different risk engines, one of them based on a general population and on a reduced number of patients with DM2 (REGICOR) and two on exclusively DM2 populations (UKPDS and ADVANCE). In the ADVANCE equation, a correction factor based on the prevalence of atrial fibrillation in the Spanish population with DM2 was considered. A patient was considered to have a high risk if the values of REGICOR ≥ 10 % after 10 years, UKPDS ≥ 15 % after 10 years, and ADVANCE ≥ 8 % after 4 years. Framingham-REGICOR provided estimates of risk for CHD, and UKPDS and ADVANCE estimate CHD and stroke (Table 1).

**Table 1:** Variables considered in each cardiovascular risk engine

	REGICOR	UKPDS	ADVANCE
Age	×	×	×
Age of DM2 diagnosis	−	×	×
Gender	×	×	×
Ethnicity	−	×	−
Tobacco	×	×	−
SBP	×	×	*
Total cholesterol	×	*	*
Time of evolution of DM2	−	×	×
HbA1c	−	×	×
Antihypertensive treatment	−	−	×
Microalbuminuria	−	−	×
Retinopathy	−	−	×
Atrial fibrillation	−	−	×

(×): included variable; (−): variable not included; (*): included variable with some modifications

*DM2* diabetes mellitus type 2, *SBP* systolic blood pressure

### Statistical analysis

The CVR was calculated using the three functions listed. The ratios were compared using the chi-square test and the means with the *t*-Student and ANOVA test or the corresponding non-parametrical tests if the conditions were not satisfied (McNemar, Mann–Whitney U and Kruskal-Wallis). An alpha level of statistical significance of < 0.05 was used in all cases. The correlation between the studied engines was analysed with the Pearson’s correlation coefficient and the Kappa coefficient considering values over 0.6 as an indicative of good agreement.

For the classification of a patient as high cardiovascular risk, the variables associated with the disagreement among the different risk functions were evaluated using generalised linear models (GLM) with disagreement (1) or no disagreement (0) as the response variables and a binomial link (logistic regressions). Due to the heterogeneity of the cohorts, the same generalised lineal model was applied separately to each cohort to confirm uniformity in the results.

All of the analyses were performed using the SPSS statistical programme version 15.0 and R free software version 3.0.1.

## Results

A total of 3,041 patients (53.8 % male; 60.4 years, standard deviation 9.7), 35 to 74 years old, both inclusive, without a previous cardiovascular disease were included. The variables analysed show significant differences in age, gender, years of diabetes diagnosed, ethnicity, tobacco consumption, pulse pressure, HbA1c, single or combined use of insulin and the prevalence of diabetic retinopathy (Table 2).

**Table 2:** Baseline characteristics of the cohorts

	All	GEDAPS 2007	IDIME	PERCEDIME2
	*n* = 3,041	*n* = 1,774	*n* = 605	*n* = 662
Age, years, mean (SD)**	60.4 (9.7)	62.4 (9.0)	53.6 (9.7)	61.3 (8.6)
Gender, men, *n* (%)*	1638 (53.8)	929 (52.3)	328 (54.2)	384 (58.0)
Years since diabetes diagnosis, mean (SD)**	6.7 (5.5)	6.3 (5.1)	6.6 (6.2)	7.8 (5.8)
Afroamericans/Afrocaribbeans, *n* (%)**	187 (14.8)		170 (28.1)	17 (2.6)
Tobacco, *n* (%)**	435 (14.3)	317 (17.8)	4 (0.6)	116 (17.5)
BMI, kg/m2, mean (SD)	30.5 (5.1)	30.5 (5.1)	29.9 (5.8)	30.5 (5.3)
Waist circumference, cm, mean (SD)*	100.9 (15.7)		102.4 (13.2)	99.7 (7.3)
Pulse pressure, mmHg, mean (SD)**	56.9 (13.1)	58.2 (12.9)	55.2 (15.1)	55.6 (11.4)
Glycated haemoglobin, %, mean (SD)**	7.1 (1.6)	6.8 (1.5)	7.6 (1.9)	7.3 (1.3)
HDL, mg/dL, mean (SD)**	48.8 (13.3)	49.8 (13.2)	47.4 (13.9)	47.9 (12.7)
Non-HDL cholesterol**	146.9 (38.3)	148.3 (37.1)	150.9 (43.7)	140.2 (35.1)
UAER, mg/gr, mean (SD)*	29.5 (109.4)	28.4 (88.2)	43.6 (185.4)	24.1 (80.4)
GFR, mil/min/1.73 m2, mean (SD)	75.6 (30.4)	76.29 (32.7)		74.1 (23.9)
Diabetic retinopathy, *n* (%)**	325 (10.7)	238 (13.4)	52 (8.6)	55 (8.3)
Diabetes therapy, *n* (%)**				
Diet	560 (18.4)	408 (23.0)	63 (10.4)	89 (13.5)
OA	1,063 (34.9)	751 (42.3)	223 (36.9)	89 (13.5)
OA + Insulin	504 (16.5)	150 (8.5)	80 (13.2)	274 (41.4)
Insulin	192 (6.3)	110 (6.2)	48 (7.9)	34 (5.1)
OA combined	722 (23.7)	355 (20.0)	191 (31.6)	176 (26.6)
Antihypertensive treatment, *n* (%)**	1837 (60.4)	1126 (63.5)	270 (44.6)	440 (66.5)
Dyslipidaemia therapy, *n* (%)**	1417 (46.6)	929 (52.4)	210 (34.8)	410 (61.9)

*BMI* body mass index, *UAER* urinary albumin excretion rate, *GFR* glomerular filtration rate according to MDRD equation, *OA* oral antidiabetic drug

*p < 0.05

**p <0.005

The ADVANCE equation identifies a greater number of diabetic patients with a high cardiovascular risk (*n* =737, 24.2 %), while REGICOR classifies the lowest number of patients with a high risk (*n* = 312, 10.2 %). The UKPDS classifies a greater number of DM2 patients with a high cardiovascular risk than the REGICOR equation, and similar to the ADVANCE equation (*n* = 691, 22.7 %).

Table 3 shows the characteristics of the DM2 patients with a high cardiovascular risk for each equation. In the ADVANCE equation, the profile of the patients with high risk are older (68.1 years; SD 5.9), with more years of evolution of DM2 (11.8; SD 7.1), a smaller number of smokers, high pulse pressure (63.9 mmHg; SD 12.8), greater impaired kidney function and retinal alterations, a higher percentage of patients treated with single or combined insulin with oral antidiabetics (OA), and a greater number of patients following an antihypertensive treatment. The UKPDS and ADVANCE equations show similar results in most of the characteristics of the high risk patients, except for age, pulse pressure, antihypertensive drug consumption, and impairment of kidney function. REGICOR classifies as high risk patients a higher number of male smokers with elevated levels of non-HDL cholesterol and low levels of HDL cholesterol.

**Table 3:** Characteristics of high-risk patients according to each risk equation

	REGICOR	UKPDS	ADVANCE
	*n* = 312	*n* = 691	*n* = 737
Age, years, mean (SD)	64.1 (7.0)	66 (6.6)	68.1 (5.9)
Gender, men, *n* (%)	221 (70.8)	469 (67.9)	451 (61.2)
Years since diabetes diagnosis, mean (SD)	7.07 (5.4)	12.9 (6.5)	11.8 (7.1)
Afroamericans/Afrocaribbeans, *n* (%)	11 (3.5)	9 (1.3)	21 (2.8)
Tobacco, *n* (%)	118 (37.8)	122 (17.7)	86 (11.7)
BMI, kg/m2, mean (SD)	30.2 (4.7)	29.9 (4.6)	30.1 (4.9)
Waist circumference, cm, mean (SD)	100.2 (19.4)	100.0 (16.7)	101.0 (15.8)
Pulse pressure, mmHg, mean (SD)	59.2 (13.0)	61.8 (13.7)	63.9 (12.8)
Glycated haemoglobin, %, mean (SD)	7.4 (1.6)	7.7 (1.6)	7.4 (1.5)
HDL, mg/dL, mean (SD)	36.5 (8.1)	45.3 (12.0)	48.6 (12.8)
Non-HDL cholesterol	171.0 (42.5)	149.7 (38.4)	140.0 (37.4)
UAER, mg/gr, mean (SD)	37.3 (94.6)	43.5 (161.7)	55.1 (150.5)
GFR, mil/min/1.73 m2, mean (SD)	44 (96)	43 (94)	50 (101)
Diabetic retinopathy, *n* (%)	22 (7.1)	98 (14.2)	140 (19.0)
Diabetes therapy, *n* (%)			
Diet	57 (18.3)	61 (8.8)	73 (10.0)
OA	99 (31.7)	185 (26.8)	232 (31.7)
OA + Insulin	66 (21.2)	152 (22.0)	143 (19.5)
Insulin	18 (5.8)	66 (9.6)	78 (10.7)
OA combined	70 (22.4)	221 (32.0)	206 (28.1)
Antihypertensive treatment, *n* (%)	226 (72.4)	491 (71.1)	589 (79.9)
Dyslipidaemia therapy, *n* (%)	168 (53.8)	375 (54.3)	421 (57.1)

High risk is defined as follows: REGICOR ≥ 10 % at 10 years; UKPDS ≥ 15 % at 10 years

ADVANCE ≥ 8 % at 4 years

*BMI* body mass index, *CBP* clinic blood pressure, *UAER* urinary albumin excretion rate, *GFR* glomerular filtration rate according to MDRD equation, *OA* oral antidiabetic drug

According to the real validity assessment, the correlation and agreement among the different equations were poor, despite the statistical significance. Only the values of correlation and agreement between the DM2 patient-specific equations (ADVANCE and UKPDS) were significant (*r* = 0.752, *p* < 0.0001 and *k* = 0.608, *p* < 0.0001; respectively). The correlation of the REGICOR equation with UKPDS and ADVANCE was low (*r* = 0.288 and *r* = 0.153, respectively; *p* < 0.0001). The agreement scores when assigning a specific patient to the high risk group were low for the REGICOR risk equation compared to UKPDS and ADVANCE (*k* = 0.205 and *k* = 0.123, respectively; *p* < 0.0001) but good when comparing the ADVANCE equation with the UKPDS equation (Table 4).

**Table 4:** Agreement among the different risk engines in the classification of high risk patients

	REGICOR	UKPDS	ADVANCE	*n*
REGICOR	1	0.205	0.123	312
		*n* = 161	*n* = 123	
UKPDS	0.205	1	0.608	691
	*n* = 161		*n* = 477	
ADVANCE	0.123	0.608	1	737
	*n* = 123	*n* = 477		
*n*	312	691	737	*n* = 1740

Index of agreement (kappa). All values are significant, *p* < 0.0001

The sensitivity, specificity and positive and negative predictive values for UKPDS and ADVANCE equations were calculated compared to REGICOR equation. For the usual cut-off points (REGICOR ≥10 %, UKPDS ≥15 %, and ADVANCE ≥8 %), and considering that REGICOR is the gold standard as the only validated engine for the Spanish population, the sensitivity was 0.518 for the UKPDS equation and 0.394 for the ADVANCE equation; the specificity was 0.798 and 0.791, respectively. The positive predictive values were 0.067 and 0.083, respectively, and the negative predictive values were 0.767 and 0.817, respectively. The positive likelihood ratios were 2.56 and 1.88, and the negative likelihood ratios were 0.6 and 0.76, respectively.

The multivariate analysis (Table 5) showed that the time of evolution of diabetes, specifically for the ADVANCE equation, the levels of HbA1c, the age of the patient, and the levels of non-HDL cholesterol were variables associated with a greater degree of disagreement between REGICOR equation and the DM2 patient-specific equations when classifying DM2 patients with a high cardiovascular risk. Other variables associated with the disagreement between REGICOR and ADVANCE equations were the presence of retinopathy, arterial hypertension with drug treatment, tobacco consumption, pulse pressure, and urinary albumin excretion. The same multivariate analysis model for each separate cohort showed similar results to the global results of the three cohorts.

**Table 5:** Multivariate analysis of variables associated with disagreement among REGICOR (Framingham adapted to the Spanish population), UKPDS, and ADVANCE functions

	REGICOR vs.	
	UKPDS (OR; CI 95 %)	ADVANCE (OR; CI 95 %)
Age centred (for year)	1.11 (1.08–1.15)*	1.42 (1.32–1.53)*
Gender (women)	0.23 (0.15–0.37)*	0.02 (0.008–0.05)*
Years since diabetes diagnosis		
6–10	2.4 (1.38–4.15)**	189.2 (47.8–748.8)*
11–20	18.1 (10.2–32.1)*	10.7 × 10^3^ (1.7 × 10^3^–65.4 × 10^3^)*
> 20	81.3 (22.2–297.6)*	2.06 × 10^5^ (8.49 × 10^3^–5.0 × 10^6^)*
Non-HDL cholesterol (mg/dL)	1.012 (1.006–1.017)*	1.02 (1.01–1.03)*
HbA1c (for each 1 %)	1.39 (1.20–1.61)*	2.68 (2.03–3.52)*
Tobacco (yes)	1.41 (0.8–2.51)	2.6 (1.04–6.53)**
UAER (mg/gr)	0.999 (0.991–1.001)	1.01 (1.00–1.02)*
Pulse pressure (mmHg)	1.01 (0.99–1.03)	1.06 (1.03–1.09)*
Hypertension treatment (yes)	1.20 (0.75–1.92)	2.98 (1.37–6.45)**
Retinopathy (yes)	1.58 (0.81–3.09)	5.42 (1.83–16.0)**

0 = no disagreement, 1 = disagreement

Adjusted for ethnicity, body mass index, waist circumference, diabetes therapy and type, systolic blood pressure, diastolic blood pressure, dyslipidaemia therapy and years since diabetes diagnosis

UAER: urinary albumin excretion rate

*p < 0.001

**p < 0.05

## Discussion

The results in this study demonstrate that the prevalence of diabetic patients with high CVR varies according to the risk equation applied. Each risk equation identifies diabetic patients with high CVR according to different profiles regarding clinical, analytical, and treatment variables. The ADVANCE equation classifies a higher number of patients with high CVR, while REGICOR classifies the smallest number with high CVR. Specifically, ADVANCE classifies 14 % more patients than REGICOR as high CVR. In addition most of the patients classified as high CVR by REGICOR equation do not match those classified as high risk by the UKPDS and ADVANCE equations, as indicated by the low agreement between the three equations. In fact, it is observed (Table 2) that high risk patients according REGICOR are smokers and have a more desvaforable lipid profile compared to patients with high risk according UKPDS and ADVANCE functions. Many of these pacients classified as high risk by REGICOR probably not fall into this category according to the UKPDS and ADVANCE functions.

A novel methodological approach in study was developed. A multivariate model was constructed and adjusted by multiple covariables to determine the disagreement between REGICOR equation and UKPDS and ADVANCE equations. In this model, the variables associated with disagreement can be identified to quantify the independent impact of each variable. In this context, some variables such as the time of evolution of diabetes, should be evaluated.

The results of the ADVANCE study [19] question the utility of the Framingham and UKPDS engines due to their low efficiency in predicting the risk of a CVD in diabetic patients. Unlike Framingham and UKPDS studies, ADVANCE study included participants from different countries and ethnicities and incorporated new predictive variables that were not featured in the Framingham and UKPDS equations. The external validation of the UKPDS engine [25] using a contemporary cohort of diabetic patients showed that the UKPDS engine has a moderate discriminative capacity and overestimates the CVR. Marrugat J et al. [26] observed that the REGICOR-adapted function’s estimate did not differ from the observed cumulated incidence. In men and women with diabetes (16.4 %), the Kaplan-Meier observed overall 5-year CHD event rate was 4.9 %. The original Framingham function significantly overestimated the event rate by a factor > 2.6; the prediction of the Framingham-REGICOR adapted function did not differ from the observed rate. Cañón Barroso L et al. [27] observed that the comparison between the mean coronary risk estimated by the original Framingham equation (Framingham-Wilson) and the calibrated one for the Spanish population (Framingham-REGICOR) in diabetic patients showed that Framingham-REGICOR underestimated the actual coronary risk (11.3 % versus 15.0 %) while Framingham-Wilson overestimated the risk (27.4 % versus 15.0 %) and Jimeno Mollet et al. [28] found that Framingham-REGICOR underestimated the coronary risk at 10 years, 20 % and 40 % for men and women respectively. European guidelines on diabetes [29] recommend not to value the risk CVD in patients with DM with risk scores developed for the general population. Framingham-REGICOR risk scores was developed for the general population, and only 16.4 % were diabetic patients, whilst in UKPDS and ADVANCE all were diabetic patients.

Sha AD et al. [30] observed that type 2 diabetes was positively associated with peripheral arterial disease (adjusted HR 2.98 [95 % CI 2.76-3.22]), ischaemic stroke (1.72 [1.52-1.95]), stable angina (1.62 [1.49-1.77]), heart failure (1.56 [1.45-1.69]), and non-fatal myocardial infarction (1.54 [1.42-1.67]). In BASCORE Study [31], in Spain, type 2 diabetes was associated with greater complications and mortality [Incidence rates (1,000 person-years)]: coronary heart disease (21.6 [95 % CI 17.9-25.9]); Stroke (14.6 [11.7-18.1]) and peripheral arterial disease (12.3 [9.6, 15.5]) and Cañón Barroso L et al. [27] found that incidence of stroke, coronary and global cardiovascular events in diabetic patients were 14.7 %, 9.3 % and 21.3 %, respectively.

When 45 prediction models were analysed, 12 of them exclusive to DM2 patients and one third of them with external validation, Van Dieren et al. indicated poor results. Furthermore, the discriminative capacity was moderate, and its use in daily clinical practice had a low impact on the final results [32].

Currently, FRAMINGHAM-REGICOR equation is recommended in Spain to estimate the coronary risk of the DM2 population (Diabetes Strategy of the National Health System 2012) [33]. The risk equations are the result of prospective cohort studies, and all estimate the probability of presenting a cardiovascular or coronary event in a determined time in the presence or absence of the CVRF. It is likely that the DM2 specific population models can predict a CVR better than the ones based on general populations as the latter tend to underestimate the risk for diabetes mellitus patients [34]. The most important aspect of a risk equation is the possibility of accurately stratifying high and low risk [35]. In addition to classical CVRF, other factors that affect the prognosis of a patient might be a family history of an early CVD, years of evolution of diabetes, high levels of HbA1c, chronic kidney disease, diabetic retinopathy and atrial fibrillation [19]. Including these factors would most likely improve the CVD stratification. Our study identifies some variables associated with the disagreement among the different equations in order to consider the development of a new DM2 population-specific risk equation: years of evolution of diabetes, pulse pressure, waist circumference, presence of kidney disease or retinopathy and treatment with insulin or antihypertensives.

Among the limitations of this study, it should be considered that this was a cross-sectional study and that morbi-mortality evolution data was not available to assess the predictive capacity of each equation. Additionally, the three cohorts included show great heterogeneity, which has prevented a bivariate analysis. However, the results of the global lineal models and the models performed identically for each cohort separately show the same results, validating the global results for all three cohorts.

## Conclusions

The practical implications of these results are evident. A further study would be necessary to carry out a new cardiovascular risk prediction model for DM2 in the Spanish (southern Europe) population. This study should focus on the validation and improvement of the performance of one of these existing prediction models, more than on the development of a new prediction model, adapting the existing model to the Spanish population and adding new predictive variables. This engine should be evaluated prospectively in a contemporary cohort of diabetic patients to provide more reliable risk estimations than the current models.

## Abbreviations

REGICOR: Girona Heart Registry. Framingham adapted to a Spanish population
UKPDS: UK prospective diabetes study
ADVANCE: Action in diabetes and vascular disease preterax and diamicron MR controlled evaluation
CVD: Cardiovascular disease
CVR: Cardiovascular risk
